# External Validation of Serologic Scores for the Detection of Liver Steatosis Among People With HIV

**DOI:** 10.1093/ofid/ofae411

**Published:** 2024-07-31

**Authors:** Carlotta Riebensahm, Julia Brocker, Annalisa Berzigotti, Huldrych F Günthard, Philip E Tarr, Hansjakob Furrer, Andri Rauch, Gilles Wandeler, Bernard Surial, Swiss HIV Cohort Study, I Abela, K Aebi-Popp, A Anagnostopoulos, M Battegay, E Bernasconi, D L Braun, H C Bucher, A Calmy, M Cavassini, A Ciuffi, G Dollenmaier, M Egger, L Elzi, J Fehr, J Fellay, H Furrer, C A Fux, H F Günthard, A Hachfeld, D Haerry, B Hasse, H H Hirsch, M Hoffmann, I Hösli, M Huber, D Jackson-Perry, C R Kahlert, O Keiser, T Klimkait, R D Kouyos, H Kovari, K Kusejko, N Labhardt, K Leuzinger, B Martinez de Tejada, C Marzolini, K J Metzner, N Müller, J Nemeth, D Nicca, J Notter, P Paioni, G Pantaleo, M Perreau, A Rauch, L Salazar-Vizcaya, P Schmid, R Speck, M Stöckle, P Tarr, A Trkola, G Wandeler, M Weisser, S Yerly

**Affiliations:** Department of Infectious Diseases, Inselspital, Bern University Hospital, University of Bern, Bern, Switzerland; Graduate School of Health Sciences, University of Bern, Bern, Switzerland; Institute of Social and Preventive Medicine, University of Bern, Bern, Switzerland; Department of Infectious Diseases, Inselspital, Bern University Hospital, University of Bern, Bern, Switzerland; Department for Visceral Surgery and Medicine, Inselspital, Bern University Hospital, University of Bern, Bern, Switzerland; Hepatology, Department of BioMedical Research, University of Bern, Bern, Switzerland; Department of Infectious Diseases and Hospital Epidemiology, University Hospital Zurich, University of Zurich, Zurich, Switzerland; Institute of Medical Virology, University of Zurich, Zurich, Switzerland; University Center for Internal Medicine, Kantonsspital Baselland, University of Basel, Bruderholz, Switzerland; Department of Infectious Diseases, Inselspital, Bern University Hospital, University of Bern, Bern, Switzerland; Department of Infectious Diseases, Inselspital, Bern University Hospital, University of Bern, Bern, Switzerland; Department of Infectious Diseases, Inselspital, Bern University Hospital, University of Bern, Bern, Switzerland; Institute of Social and Preventive Medicine, University of Bern, Bern, Switzerland; Department of Infectious Diseases, Inselspital, Bern University Hospital, University of Bern, Bern, Switzerland

**Keywords:** external validation, liver steatosis, metabolic dysfunction–associated steatotic liver disease, people with HIV, serologic scores

## Abstract

**Background:**

Fatty liver index (FLI) and hepatic steatosis index (HSI) are serologic scores used to detect liver steatosis. However, their diagnostic performance in people with HIV (PWH) remains unclear. We performed an external validation of FLI and HSI in the Swiss HIV Cohort Study.

**Methods:**

We systematically performed vibration-controlled transient elastography (VCTE) among Swiss HIV Cohort Study participants at Bern University Hospital between November 2019 and August 2021. Individuals with viral hepatitis and pregnant women were excluded. We defined liver steatosis as controlled attenuation parameter ≥248 dB/m using VCTE. Model discrimination was assessed with the C-index and model calibration with calibration plots. A decision curve analysis was performed to compare the clinical usefulness of both scores.

**Results:**

Of 321 participants, 91 (28.4%) were female, the median age was 51.4 years (IQR, 42–59), 230 (71.7%) were Caucasian, and 164 (51.1%) had a body mass index >25 kg/m^2^. VCTE-confirmed liver steatosis was present in 158 (49.2%). Overall, 125 (38.9%) had an FLI ≥60, and 128 (39.9%) had an HSI ≥36. At these cutoffs, the C-index to diagnose liver steatosis was 0.85 for FLI (95% CI, .80–.89) and 0.78 for HSI (95% CI, .73–.83). Whereas FLI was well calibrated, HSI overestimated the risk for steatosis. Both models showed a positive net benefit, with FLI having a greater net benefit when compared with HSI.

**Conclusions:**

FLI and HSI are valid tools to detect liver steatosis in PWH. FLI should be the preferred score, given its better performance and greater clinical usefulness.

## Graphical Abstract

**Graphical Abstract:**
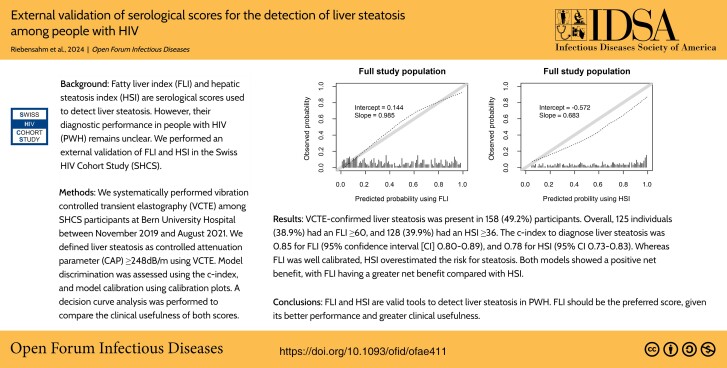
This graphical abstract is also available at Tidbit: https://tidbitapp.io/tidbits/external-validation-of-serological-scores-for-the-detection-of-liver-steatosis-among-people-with-hiv-open-forum-infectious-diseases-6598b433-21db-43bb-881d-508733d014b8

## Teaser

Liver steatosis is common in people with HIV. Fatty liver index and hepatic steatosis index are valid scores based on clinical and laboratory variables to detect liver steatosis in people with HIV and allow a targeted approach to screening.

## BACKGROUND

Liver steatosis can lead to steatohepatitis and progress to liver fibrosis and cirrhosis [1, 2]. Liver steatosis is highly prevalent and seems to progress more rapidly among people with HIV (PWH) as compared with the general population [3, 4]. Furthermore, liver steatosis is associated with cardiovascular disease, with cardiovascular events being the leading cause of mortality in patients with steatosis [5, 6]. Given these concerns, identifying PWH with liver steatosis is crucial for further assessment, to promote lifestyle interventions, and to optimize the management of cardiometabolic conditions [7, 8].

The European AIDS Clinical Society recommends assessing and monitoring liver disease severity in PWH in case of suspected liver steatosis and metabolic risk factors using abdominal ultrasound or vibration-controlled transient elastography (VCTE) [9]. As access to VCTE is limited in routine HIV care, screening uptake remains limited.

Two liver steatosis prediction tools could help clinicians in deciding whether a patient should undergo further diagnostic assessment: (1) hepatic steatosis index (HSI), which is based on alanine aminotransferase, aspartate aminotransferase, body mass index (BMI), diabetes, and sex and was derived from a Korean cohort undergoing a health check-up; (2) fatty liver index (FLI), which is based on BMI, waist circumference, gamma-glutamyl transferase, and triglycerides and was developed in a sample with suspected liver disease and matched controls in Italy [10, 11]. Both scores have been validated for the detection of liver steatosis in the general population and patients with diabetes. However, their diagnostic accuracy and clinical utility remain incompletely understood among PWH, as few studies have validated the two scores’ calibration and only one study assessed their clinical utility [12–15].

Considering that the prevalence of steatosis and related conditions such as diabetes is higher among PWH as compared with the general population, and that antiretroviral therapy affects body weight, dedicated validation of the scores is needed for PWH [4, 16, 17]. We conducted an external validation of HSI and FLI to evaluate their diagnostic accuracy, model calibration, and net benefit for the detection of liver steatosis among PWH at a single center of the Swiss HIV Cohort Study (SHCS).

## METHODS

### Study Design and Population

Between November 2019 and August 2021, we systematically assessed liver steatosis among SHCS participants at Bern University Hospital using VCTE. PWH with VCTE and available data for the calculation of HSI and FLI were included in this study. The study excluded individuals with active or past viral hepatitis, defined as having a detectable hepatitis B virus surface antigen or positive hepatitis C virus antibody test result, and pregnant women. The SHCS (www.shcs.ch) is a nationally representative prospective cohort, including close to 80% of all PWH currently receiving antiretroviral therapy in Switzerland [18]. Laboratory values, sociodemographic and clinical data, anthropometric measurements, and data on antiretroviral therapy regimens are prospectively recorded at 6-month intervals via a standardized protocol (http://shcs.ch/292-instructions). Plasma samples were stored within a window of seven days before or after each VCTE measurement. This study follows the TRIPOD guidelines (Transparent Reporting of a Multivariable Prediction Model for Individual Prognosis or Diagnosis) and STARD statement (Standards for Reporting of Diagnostic Accuracy Studies) [19, 20].

### Outcomes and Definitions

We evaluated the performance of FLI and HSI for the diagnosis of liver steatosis, using VCTE as the reference. VCTE (FibroScan 530; Echosens) was performed by two experienced operators (C. R. and J. B.) to determine liver stiffness measurement (LSM) and the controlled attenuation parameter (CAP). A minimum of 10 valid measurements with a success rate >60% was required [21]. Probe selection was made by the automatic selection tool embedded in the device software. Liver steatosis was defined as follows: with the M probe, S1 (mild steatosis) if the CAP was 248 to 267 dB/m, S2 (moderate steatosis) if 268 to 279 dB/m, and S3 (severe steatosis) if ≥280 dB/m; with the XL probe, S1 if 242 to 266 dB/m, S2 if 267 to 285 dB/m, and S3 if ≥286 dB/m [22–24]. Liver fibrosis was categorized according to METAVIR-equivalent stages: F0 or F1 (no or mild fibrosis) if the LSM was <7.1 kPa, F2 or F3 (significant fibrosis) if 7.1 to 11 kPa, and F4 (cirrhosis) if ≥11.1 kPa, as established previously [25]. In addition, we calculated the FibroScan-AST (FAST) score, which is based on LSM, CAP, and aspartate aminotransferase. A FAST score >0.67 indicated steatohepatitis with significant fibrosis [26].

In accordance with the original publications, HSI and FLI were calculated as follows, based on laboratory values obtained on the same day or within a period of ±7 days from the time of VCTE [10, 11]:


$$\begin{array}{c} \mathrm{FLI}:\frac{\left(e^{0.953\times \mathrm{loge}(\mathrm{triglycerides})+0.139*\mathrm{BMI}+0.718\times \mathrm{loge}(\mathrm{ggt})\;+\;0.053\;\times \;\mathrm{waist}\;\mathrm{circumference}-15.745}\right)}{(1+e^{0.953\times \mathrm{loge}\;(\mathrm{triglycerides})+0.139\times \mathrm{BMI}+0.718\times \mathrm{loge}(\mathrm{ggt})+0.053\times \mathrm{waist}\;\mathrm{circumference}-15.745})\;\times \;100}\\ \mathrm{HSI}:8\;\times \;(\mathrm{ALT}/\mathrm{AST})+\mathrm{BMI}(+2,\mathrm{if}\;\mathrm{type}\;2\;\mathrm{diabetes};+2,\;\mathrm{if}\;\mathrm{female}) \end{array}$$

If laboratory values for calculating the scores were not available in the SHCS, we used the stored sample to determine missing values retrospectively.

Participants with a BMI <25 kg/m^2^ were categorized as lean and those with a value ≥25 kg/m^2^ as overweight or obese. Hypertension was defined as two measurements ≥140/90 mm Hg within one year prior to VCTE or current antihypertensive treatment; diabetes as hemoglobin A_1c_ ≥6.5% or current treatment with antidiabetic medication; and dyslipidemia as a total cholesterol:high-density lipoprotein ratio >5 or current receipt of a lipid-lowering therapy. A history of cardiovascular disease included myocardial infarction, cerebral infarction, coronary angioplasty/stenting, coronary artery bypass grafting, or any procedure on peripheral arteries [18]. Hazardous alcohol consumption was defined as an Alcohol Use Disorders Identification Test (AUDIT-C) score ≥4 for men and ≥3 for women [27].

### Statistical Analyses

We assessed the performance of FLI and HSI by examining measures of discrimination and calibration. Discrimination is the ability of a score to distinguish individuals at high risk from those of low risk, and it was assessed by calculating the C-index, which is equivalent to the area under the receiver operating characteristic curve [28]. In addition, we calculated the sensitivity, specificity, negative predictive values (NPV), positive predictive values (PPV), positive likelihood ratios, and negative likelihood ratios at previously validated thresholds for steatosis (≥36 for HSI and ≥60 for FLI) [10, 11].

Calibration indicates the agreement between the predicted risks and the observed frequencies of the outcome, and it was evaluated by using calibration plots and calculating the calibration slope and intercept [29, 30]. The calibration plot compares the predicted risk based on the model (x-axis) with the observed proportion of events in the study sample (y-axis). A diagonal straight line indicates perfect agreement between predicted and observed probabilities, which is equivalent to perfect model calibration. If the line deviates below the ideal diagonal line, the model overestimates the actual risk of the outcome. In contrast, a line that lies above the ideal diagonal one indicates underestimation. The calibration slope and intercept evaluate the spread of the predicted risks on average: a slope of 1 and an intercept of 0 would indicate a perfect calibration. A slope <1 or a negative intercept suggests that predicted risks are too extreme, and a slope >1 or a positive intercept indicates that risk estimates are too moderate [30].

We used decision curve analyses to demonstrate and compare the clinical usefulness of both scores by comparing their net benefit against the strategy “test all” or “test none” [31, 32]. The net benefit is calculated for a range of sensible threshold probabilities. The net benefit describes the number of true positives identified, adjusted for the impact of false positives. For example, a net benefit of 0.2 indicates that if we test 100 individuals, the benefit is equivalent to correctly identifying 20 true positives after accounting for the harm of false positives. The threshold probability is a subjective measure and expresses the probability of the disease at which a next test would be acceptable. For example, a threshold probability of 25% indicates that one would proceed to VCTE or ultrasound if the probability of liver steatosis is ≥25%. In contrast to accuracy measures such as discrimination and calibration, it incorporates the consequences of the clinical decision made. A model with a higher net benefit identifies more true positives than a model with a lower net benefit. Therefore, a test is considered to have clinical value if it has the highest net benefit across a large range of threshold probabilities. All statistical analyses were performed with Stata version 16.0 (StataCorp) and R (version 4.3).

### Subgroup and Sensitivity Analyses

We evaluated measures of discrimination and calibration among the full study population and as stratified by sex (women, men) and age group (<50 vs ≥50 years). As some studies suggest a higher cutoff to define steatosis among PWH, we performed a sensitivity analysis repeating the analyses for the outcome of severe liver steatosis among the full study population [33].

### Patient Consent Statement

The local ethical committee of the participating center (Kantonale Ethikkommission Bern) approved the cohort study, and all patients provided written informed consent.

## RESULTS

### Study Population

Between November 2019 and August 2021, 418 SHCS participants underwent VCTE. We excluded 95 participants (22.7%) without data on serologic scores and two patients (0.5%) with an unreliable VCTE measurement (Supplementary Figure 1). Of 321 individuals, 91 (28.5%) were female, 230 (71.7%) were Caucasian, and 64 (19.9%) were of African origin. At the time of VCTE, the median age was 51.4 years (IQR, 42–59), 164 (51.1%) were overweight or obese, 117 (36.5%) had dyslipidemia, 92 (28.6%) had hypertension, 26 (8.1%) had diabetes, and 66 (22.8%) reported hazardous alcohol consumption (Table 1). As compared with the original derivation study for FLI, participants in the present study were younger (median age, 51 years in our study vs 58 years in the derivation study), were more likely to be male (72% vs 61%), and had a lower median BMI (25.2 vs 29.5 kg/m²). Demographic and clinical characteristics in the original derivation study for HSI were similar to the present study. However, patient information on confirmed diagnoses of cardiometabolic outcomes and the presence of significant liver fibrosis was not reported in the derivation studies.

**Table 1.** Demographic and Clinical Characteristics of Participants at the Time of VCTE Measurement (N = 321)

Characteristic	Median (IQR) or No. (%)
Age, y	51 (42–59)
Female sex	91 (28.4)
Region of origin	
Europe	230 (71.7)
Africa	64 (19.9)
Asia	18 (5.6)
Hispano-American	8 (2.5)
Other/unknown	1 (0.3)
HIV transmission group	
Men who have sex with men	152 (47.4)
Heterosexual	147 (45.8)
Persons who inject drugs	1 (0.3)
Other/unknown	21 (6.5)
Overweight or obese	164 (51.1)
Hypertension	92 (28.6)
History of cardiovascular disease	21 (6.5)
Diabetes	26 (8.1)
Dyslipidemia	117 (36.5)
ALT, U/L	29.1 (21–35)
Triglycerides, mmol/L	1.9 (1.0–2.2)
Hazardous alcohol consumption	66 (22.8)
CD4+ count, cells/µL	718 (546–933)
CD4+ nadir, cells/µL	229 (118–355)
Time on ART, y	12.3 (6–19)
Current ART regimen	
3TC/ABC/DTG	73 (22.7)
FTC/TAF/BIC	43 (13.4)
FTC/TAF/DTG	46 (14.3)
FTC/TAF/COB/DRV	20 (6.2)
FTC/TAF/COB/EVG	19 (5.9)
FTC/TAF/RPV	12 (3.7)
Other	103 (32.1)
No treatment	5 (1.6)
Liver steatosis grade (CAP)	
S0 (<248 dB/m)	163 (50.8)
S1 (248–267 dB/m)	27 (8.4)
S2 (268–279 dB/m)	24 (7.5)
S3 (≥280 dB/m)	107 (33.3)
Steatohepatitis with significant fibrosis by FAST score	3 (0.9)

Abbreviations: 3TC, lamivudine; ABC, abacavir; ALT, alanine aminotransferase; ART, antiretroviral therapy; AST, aspartate aminotransferase; BIC, bictegravir; CAP, controlled attenuation parameter, determined using vibration-controlled transient elastography; COB, cobicistat; DRV, darunavir; DTG, dolutegravir; EVG, elvitegravir; FAST, FibroScan-AST score; FTC, emtricitabine; RPV, rilpivirine; TAF, tenofovir alafenamide.

VCTE-confirmed liver steatosis was diagnosed in 158 participants (49.2%): 27 (8.4%) had mild liver steatosis based on CAP, 24 (7.5%) had moderate, and 107 (33.3%) had severe steatosis. Among individuals with liver steatosis, 4 (1.3%) had an LSM compatible with significant fibrosis (F2 or F3), 3 (0.9%) had a LSM compatible with cirrhosis (F4), and 3 (0.9%) had a FAST score compatible with steatohepatitis and significant fibrosis (Table 1). The prevalence of liver steatosis was 40.7% in women and 52.6% in men. Among participants aged <50 years, 36.1% had liver steatosis, as opposed to 50.9% of those aged ≥50 years.

### External Validation of FLI and HSI

Based on the preestablished cutoff ≥60, FLI showed good discrimination in the full study population with a C-index of 0.85 (95% CI, .80−.89), which was similar to the results of the original FLI derivation study (C-index, 0.84; Supplementary Figure 2) [11]. The C-index was >0.75 in all subgroups and highest in participants <50 years old (C-index, 0.90; 95% CI, .86–.95). The overall C-index for HSI was 0.78 (95% CI, .73–.83) and highest among women (C-index, 0.85; 95% CI, .77–.94) and individuals <50 years old (C-index, 0.85; 95% CI, .78–.91). The sensitivity, specificity, NPV, and PPV overall and stratified by sex and age are summarized in Table 2.

**Table 2.** Accuracy of Serologic Scores for Detection of Liver Steatosis Among the Full Study Population and by Sex and Age

Cohort	C-index	Sensitivity, %	Specificity, %	PPV, %	NPV, %	LR+	LR−
Total (N = 321)							
HSI ≥36	0.78 (.73–.83)	61.4 (53.3–69.0)	81.0 (74.1–86.7)	75.8 (67.4–82.9)	68.4 (61.3–74.9)	3.23 (2.30–4.54)	0.48 (.39–.59)
FLI ≥60	0.85 (.80–.89)	63.3 (55.3–70.8)	84.7 (78.2–89.8)	80.0 (71.9–86.6)	70.4 (63.5–76.7)	4.13 (2.82–6.03)	0.43 (.35–.54)
Women (n = 91)							
HSI ≥36	0.85 (.77–.94)	83.8 (68–93.8)	75.9 (62.4–86.5)	70.5 (54.8–83.2)	87.2 (74.3–95.2)	3.48 (212–5.71)	0.21 (.10–.45)
FLI ≥60	0.87 (.80–.94)	67.6 (50.2–82.0)	88.9 (77.4–95.8)	80.6 (62.5–92.5)	80.0 (67.7–89.2)	6.08 (2.77–13.4)	0.36 (.23–.59)
Men (n = 230)							
HSI ≥36	0.77 (.71–.83)	54.5 (45.2–63.6)	83.5 (75.2–89.9)	78.6 (68.3–86.8)	62.3 (53.9–70.2)	3.3 (2.10–5.19)	0.54 (.44–.67)
FLI ≥60	0.84 (.78–.89)	62.0 (52.7–70.7)	82.6 (74.1–89.2)	79.8 (70.2–87.4)	66.2 (67.6–74.1)	3.56 (2.31–5.48)	0.46 (.36–.59)
Age <50 y (n = 144)							
HSI ≥36	0.85 (.78–.91)	75.0 (61.1–86.0)	81.5 (72.1–88.9)	69.6 (55.9–81.2)	85.2 (76.1–91.9)	4.06 (2.57–6.41)	0.31 (.19–.50)
FLI ≥60	0.90 (.86–.95)	65.4 (50.9–78.0)	89.1 (80.9–94.7)	77.3 (62.2–88.5)	82.0 (73.1–89.0)	6.02 (3.2–11.2)	0.39 (.3–.6)
Age ≥50 y (n = 177)							
HSI ≥36	0.76 (.68–.83)	54.7 (44.8–64.4)	80.3 (69.1–88.8)	80.6 (69.5–88.9)	54.3 (44.3–64.0)	2.77 (1.68–4.58)	0.56 (.44–.72)
FLI ≥60	0.79 (.71–.86)	62.3 (52.3–71.5)	78.9 (67.6–87.7)	81.5 (71.3–89.2)	58.3 (47.8–68.3)	2.95 (1.84–4.73)	0.48 (.36–.63)

Serologic scores are based on preestablished cutoffs: ≥60 for FLI and ≥36 for HSI [10, 11]. Data in parentheses are 95% confidence intervals.

Abbreviations: FLI, fatty liver index; HSI, hepatic steatosis index; LR+, positive likelihood ratio; LR–, negative likelihood ratio; NPV, negative predictive value; PPV, positive predictive value.

**Figure 1.**
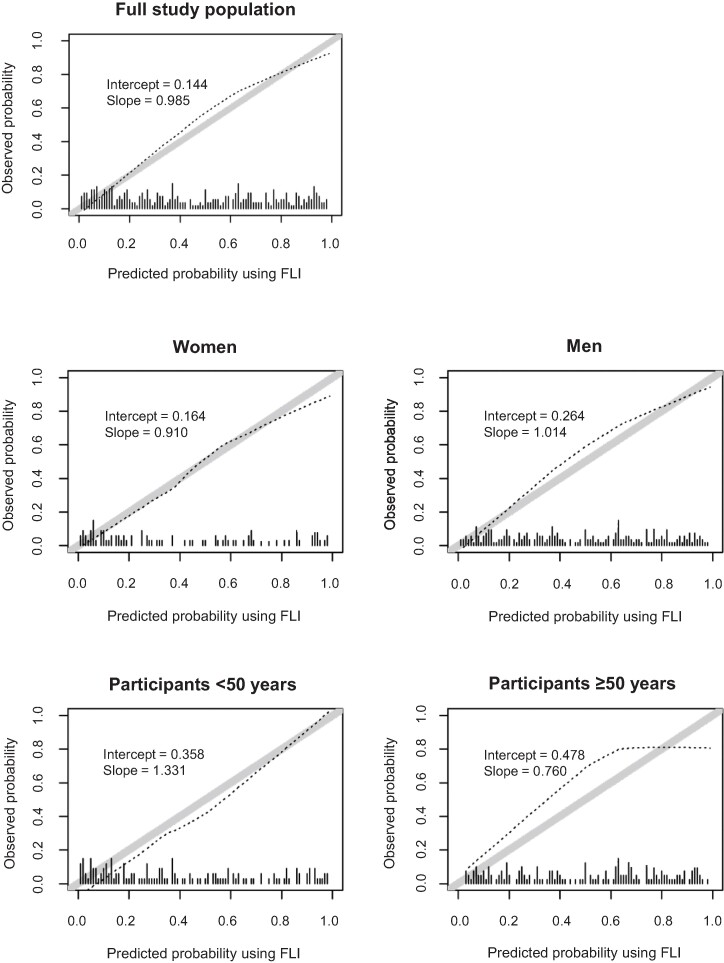
Predicted vs observed probability of liver steatosis per the FLI in the full study population, by sex and age group. The diagonal gray solid line indicates perfect agreement between predicted and observed probabilities. If the dashed black line deviates below the diagonal gray line, the model overestimates the risk of the outcome; if the dashed black line lies above the gray solid line, the model underestimates the risk. The histogram along the x-axis (small vertical lines) represents the distribution of the predicted probabilities. FLI, fatty liver index.

The calibration plots for FLI showed good agreement between the risk observed with VCTE and the risk predicted by the model across the whole range of probabilities among the full study population (calibration slope, 0.99; intercept, 0.14). In subgroup analyses, the score showed good calibration among women, men, and participants aged <50 years but tended to underestimate the risk for liver steatosis in participants aged ≥50 years when predicting low and medium risk (Figure 1).

**Figure 2.**
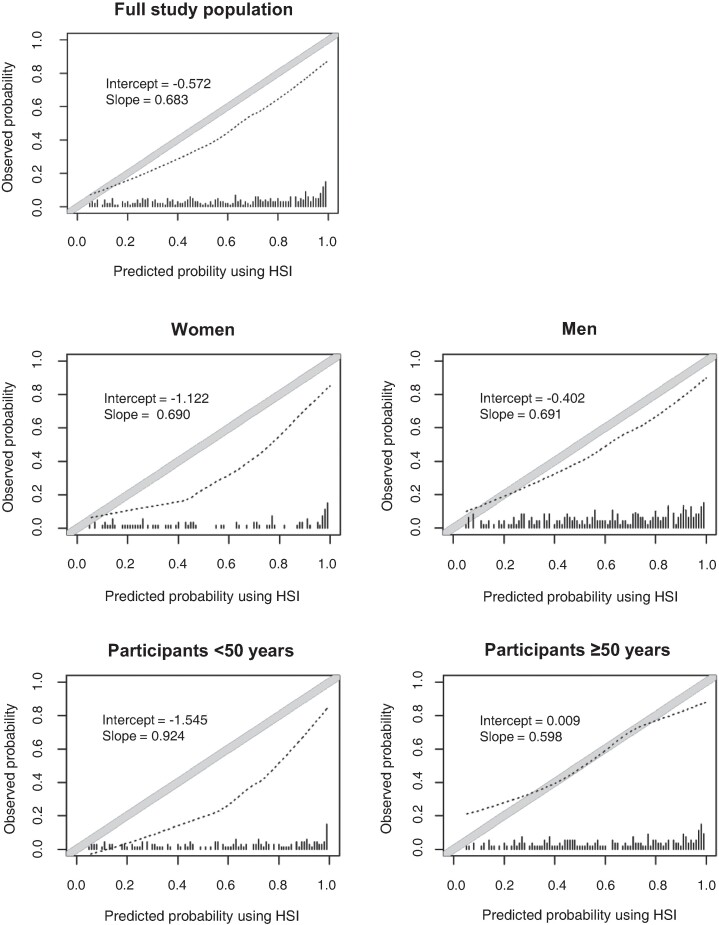
Predicted vs observed probability of liver steatosis per the HSI in the full study population, by sex and age group. The diagonal gray solid line indicates perfect agreement between predicted and observed probabilities. If the dashed black line deviates below the diagonal gray line, the model overestimates the risk of the outcome; if the dashed black line lies above the gray solid line, the model underestimates the risk. The histogram along the x-axis (small vertical lines) represents the distribution of the predicted probabilities. HSI, hepatic steatosis index.

The calibration plot for HSI indicated that the score overestimated the risk for liver steatosis among all probability thresholds by about 10% for the full study population (calibration slope, 0.68; intercept, −0.57) and among subgroups. Miscalibration was most notable when we restricted the analyses to women and to individuals <50 years old. Among participants ≥50 years, the calibration plot showed that the predicted prevalence of liver steatosis was underestimated at the lower range of estimated risks and overestimated at the higher range (Figure 2).

**Figure 3.**
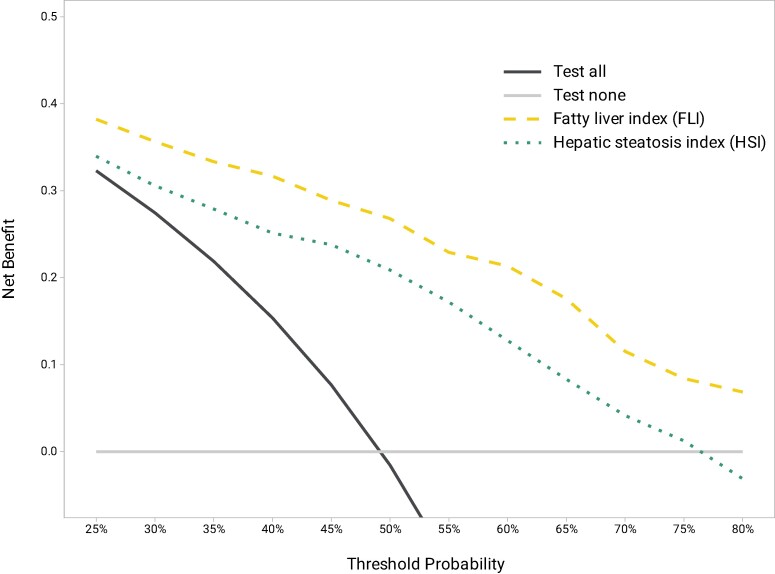
Decision curve analysis of the two prediction models for liver steatosis in the full study population. The decision curve helps to quantify the clinical impact of using a prediction model. To do so, the net benefit is calculated across a range of threshold probabilities. The net benefit describes the number of true positives identified, adjusted for the impact of false positives. For example, a net benefit of 0.2 indicates that if we test 100 individuals, the benefit is equivalent to correctly identifying 20 true positives after accounting for the harm of false positives. The threshold probability is a subjective measure and expresses the probability of the disease at which a next test would be acceptable. For example, a threshold probability of 25% indicates that one would proceed to VCTE or ultrasound if the probability of liver steatosis is ≥25%. If VCTE or ultrasound is readily available on-site, such a low threshold probability may be adequate to minimize the number of false-negative results. However, if patients need to be referred to a specialist to have VCTE performed, higher threshold probabilities (eg, 40%–50%) may be more reasonable. The present decision curve indicates that between a probability threshold of 25% and 80%, the FLI (dashed yellow line) provides a higher net benefit when compared with the HSI (dotted green line). However, both scores are superior to a strategy of performing unguided screening with VCTE for all individuals (solid black line). FLI, fatty liver index; HSI, hepatic steatosis index; VCTE, vibration-controlled transient elastography.

Figure 3 displays the net benefit curves for FLI and HSI. Both models showed a positive net benefit, meaning a greater net benefit across all risk probabilities than the alternative strategies: test all or test no one. However, FLI provided a higher net benefit than HSI across the entire range of threshold probabilities.

### Sensitivity Analyses

In sensitivity analyses for the outcome of severe liver steatosis, the scores showed similar discrimination, with a C-index of 0.83 (95% CI, .79–.88) for FLI and 0.80 (95% CI, .75–.85) for HSI and with an NPV of 84.7% (95% CI, 78.9%–89.4%) and 83.4% (95% CI, 77.4%–88.4%), respectively (Supplementary Table 1). However, calibration plots indicated that both scores overestimated the risk of severe liver steatosis (Supplementary Figure 3).

## DISCUSSION

In this external validation study, FLI and HSI both showed good accuracy in detecting liver steatosis among PWH in Switzerland, a frequent condition in this patient population. While FLI was well calibrated, HSI overestimated the risk for liver steatosis. Overall, FLI had a higher net benefit and showed better clinical usefulness than HSI.

Our findings confirm and extend previous studies that validated the two scores in PWH and found higher C-indexes and an overall better performance for FLI as compared with HSI [12, 14, 34, 35]. Likewise, in our study, model discrimination for FLI was comparable to the original derivation study, whereas HSI had an inferior discriminative ability [10, 11]. Among the few validation studies of PWH, a study from Brazil found a better discriminative ability for both scores as compared with other studies, with a C-index of 0.84 for FLI and 0.81 for HSI [12]. In contrast, a European multicenter study among PWH with metabolic syndrome or persistently elevated liver enzymes and/or clinical lipodystrophy showed poor discrimination for FLI, with a C-index of 0.69 [36].

No external validation among PWH and only a few studies in the general population have previously assessed measures of calibration and the clinical utility of the scores, which is problematic since poor model calibration may lead to potentially harmful clinical decisions [30]. In our study, HSI tended to overestimate the risk of steatosis in the full study population as well as in subgroups, whereas FLI showed good calibration, resulting in a greater net benefit across the entire range of threshold probabilities. These discrepancies in model calibration for HSI may be explained by differences in the study populations, as our participants were primarily of European origin, whereas participants in the derivation study were from Korea. Specifically, the association between BMI and cardiometabolic outcomes differs among ethnic groups, with negative health outcomes occurring at a lower BMI in Asian populations as compared with other individuals [37]. Furthermore, specific variables included in the FLI, such as triglycerides, could have a better predictive value. In addition, FLI and HSI were derived via abdominal ultrasound to diagnose liver steatosis, which is operator dependent and less sensitive for mild or moderate steatosis when compared with the VCTE used in our study [38]. Therefore, our study population was more likely than the derivation studies to include participants with mild and moderate steatosis, which may have further affected model calibration of HSI [39].

Current guidelines recommend assessing liver steatosis and monitoring disease severity in the general population as well as in PWH to prevent further complications [8, 9]. Although neither HSI nor FLI is an ideal diagnostic test due to each index’s modest NPV and PPV, options for screening large numbers of individuals via ultrasound and VCTE are limited due to their high cost and restricted availability. Our decision curve analysis shows that performing one of the two scores prior to proceeding to VCTE or ultrasound has a higher net benefit when compared with screening all PWH. Since 61.1% and 60.1% of individuals in our study had an FLI <60% and an HSI <36, respectively, targeting screening efforts to those with an FLI or HSI above the established cutoff would substantially reduce the need for screening all PWH. Patients with an FLI or HSI above the established cutoff should be actively screened for cardiometabolic conditions, offered lifestyle interventions, and screened for liver fibrosis according to existing guidelines [8]. Although FLI showed a higher net benefit than HSI, FLI includes variables that may be less accessible in certain settings. Therefore, updating the HSI model through recalibration and by extension with additional readily available covariates could be considered a next step to improve its predictive performance [40].

We performed an external validation of HSI and FLI in a well-characterized and representative cohort of PWH in Switzerland who were systematically screened by VCTE. Only two experienced operators performed the VCTE to ensure consistency of the results. Anthropometric measures and blood samples were measured on the same day or within ±seven days from the time of VCTE. We used a systematic approach to model validation, including the assessment of model discrimination, calibration, and calculation of the net benefit based on decision curve analyses [41]. However, we acknowledge a relatively low number of PWH of African origin in our study, limiting the generalizability to other settings. Subgroup analyses were based on small numbers, which limited our ability to draw strong conclusions. Finally, liver steatosis was determined by VCTE rather than ultrasound, the diagnostic method of choice to date. Yet, VCTE has become a valid alternative as it provides consistent results, has high sensitivity for liver steatosis and simultaneously provides information on liver fibrosis.

In conclusion, our results confirm that FLI and HSI are simple and valid prediction tools to identify PWH at risk of liver steatosis and to determine the need of further assessment and clinical management. These scores represent an effective alternative to screening all PWH with VCTE or ultrasound and enable larger epidemiologic studies to determine the prevalence and evolution of liver steatosis. Using these tools can increase screening and surveillance in PWH, decrease liver-related complications, and reduce imaging tests for those at low risk of liver steatosis.

## Supplementary Data

Supplementary materials are available at *Open Forum Infectious Diseases* online. Consisting of data provided by the authors to benefit the reader, the posted materials are not copyedited and are the sole responsibility of the authors, so questions or comments should be addressed to the corresponding author.

## Supplementary Material

ofae411_Supplementary_Data

## Notes

***Acknowledgments.*** We thank all patients, doctors, and nurses associated with the SHCS: I. Abela, K. Aebi-Popp, A. Anagnostopoulos, M. Battegay, E. Bernasconi, D. L. Braun, H. C. Bucher, A. Calmy, M. Cavassini, A. Ciuffi, G. Dollenmaier, M. Egger, L. Elzi, J. Fehr, J. Fellay, H. Furrer, C. A. Fux, H. F. Günthard (president of the SHCS), A. Hachfeld, D. Haerry (deputy of Positive Council), B. Hasse, H. H. Hirsch, M. Hoffmann, I. Hösli, M. Huber, D. Jackson-Perry (patient representatives), C. R. Kahlert (chair of the Mother and Child Substudy), O. Keiser, T. Klimkait, R. D. Kouyos, H. Kovari, K. Kusejko (head of Data Centre), N. Labhardt, K. Leuzinger, B. Martinez de Tejada, C. Marzolini, K. J. Metzner, N. Müller, J. Nemeth, D. Nicca, J. Notter, P. Paioni, G. Pantaleo, M. Perreau, A. Rauch (chair of the Scientific Board), L. Salazar-Vizcaya, P. Schmid, R. Speck, M. Stöckle (chair of the Clinical and Laboratory Committee), P. Tarr, A. Trkola, G. Wandeler, M. Weisser, S. Yerly.

***Author contributions*.** C. R., A. R., G. W., and B. S. designed the study. C. R., J. B., and B. S. drafted the first version of the manuscript. C. R., J. B., and B. S. performed the statistical analyses. A. B., H. F. G., P. E. T., and H. F. contributed to the conception of the study and revised the manuscript for substantial intellectual content. All authors read and approved the final manuscript.

***Financial support***. This work was supported by the framework of the Swiss HIV Cohort Study, which is funded by the Swiss National Science Foundation (grant 201369, SHCS project 861) and supported by the Swiss HIV Cohort Study Research Foundation. G. W. was supported by a professorship from the Swiss National Science Foundation (PP00P3_211025); and C. R. by the Protected Research Time Grant for PhD students of the University of Bern.
