# Supplementary material for: External Validation of Serologic Scores for the Detection of Liver Steatosis Among People With HIV
Source: Open Forum Infect Dis. 2024 Jul 31;11(9):ofae411. doi: 10.1093/ofid/ofae411 (PMC11398894; doi:10.1093/ofid/ofae411)

Supplementary material

Figure S1. Study flow chart.
VCTE, vibration-controlled transient elastography; FLI, fatty liver index; HSI, hepatic steatosis index.

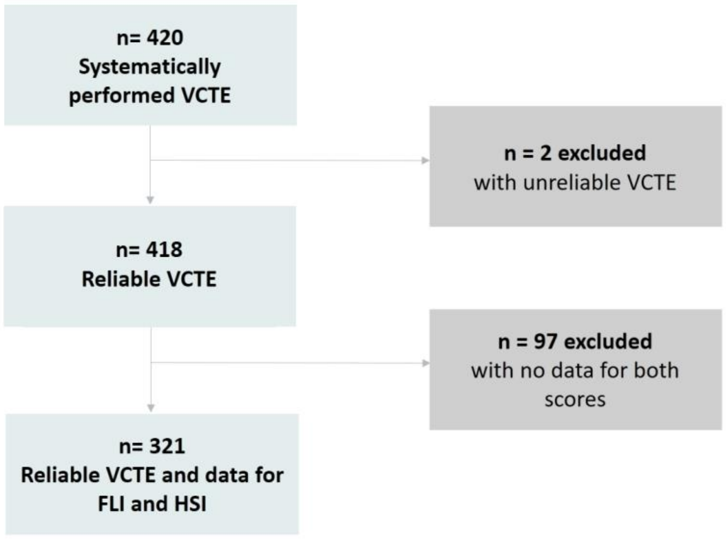

Figure S2. Receiver operator characteristic (ROC) curve for FLI (solid line) and HSI (dashed line) for the detection of liver steatosis (S 1-3) in the full study population, by sex and age group.
Numbers represent the area under the curve (AUC) measures for steatosis classification.
FLI, fatty liver index, HSI, hepatic steatosis index.

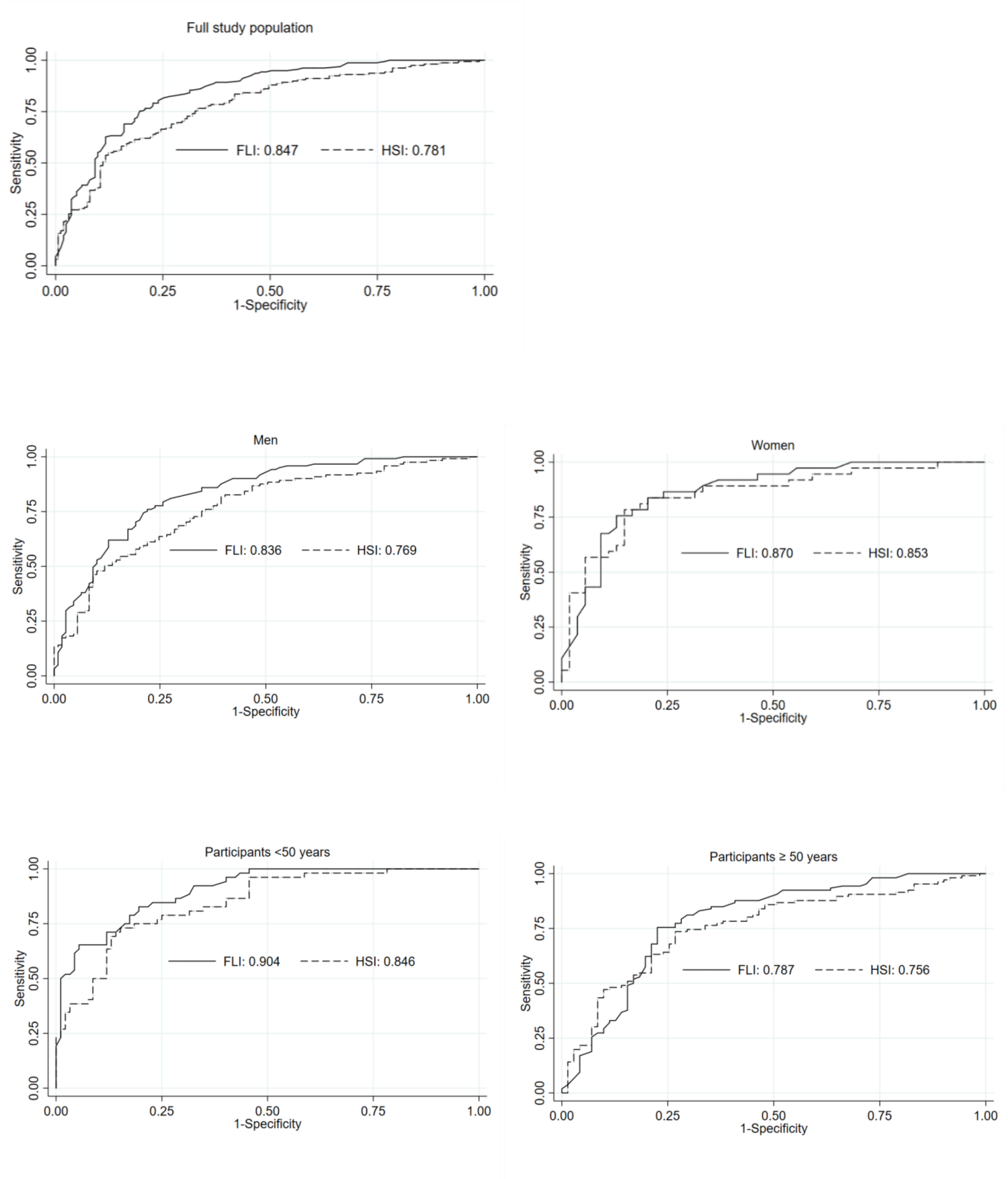

Table S1. Accuracy of serological scores for detection of severe liver steatosis (CAP ≥280dB/m) among the full study population. CI, confidence interval; FLI, fatty liver index; HSI, hepatic steatosis index; LR+, positive likelihood ratio ; LR- negative likelihood ratio; n, number; NPV, negative predictive value; PPV, positive predictive value; Sens, sensitivity; Spec, specificity.

Score	c-index (95% CI)	Sens % (95% CI)	Spec % (95% CI)	PPV % (95% CI)	NPV % (95% CI)	LR + (95% CI)	LR - (95% CI)
FLI ≥ 60	**0.83** (0.79-0.88)	**72.0** (62.5-80.2)	**77.6** (71.4-83.0)	**61.6** (52.5-70.2)	**84.7** (78.9-89.4)	**3.21** (2.44-4.23)	**0.36** (0.26-0.49)
HSI ≥ 36	**0.80** (0.75-0.85)	**70.1** (60.5-78.6)	**75.2** (68.9-80.9)	**58.6** (49.6-67.2)	**83.4** (77.4-88.4)	**2.83** (2.17-3.69)	**0.40** (0.29-0.54)

*based on pre-established cut-offs of ≥60 for FLI and ≥36 for HSI [10, 11]

Figure S3. Predicted versus observed probability of severe liver steatosis (CAP ≥280dB/m) using a) FLI and b) HSI in the full study population group. The diagonal grey solid line indicates perfect agreement between predicted and observed probabilities. If the dashed black line deviates below the diagonal grey line, the model overestimates the risk of the outcome; if the dashed black line lies above the grey solid line one, the model underestimates the risk. The histogram along the x-axis (small vertical lines) represent the distribution of the predicted probabilities. FLI, Fatty Liver Index, HSI, Hepatic Steatosis Index

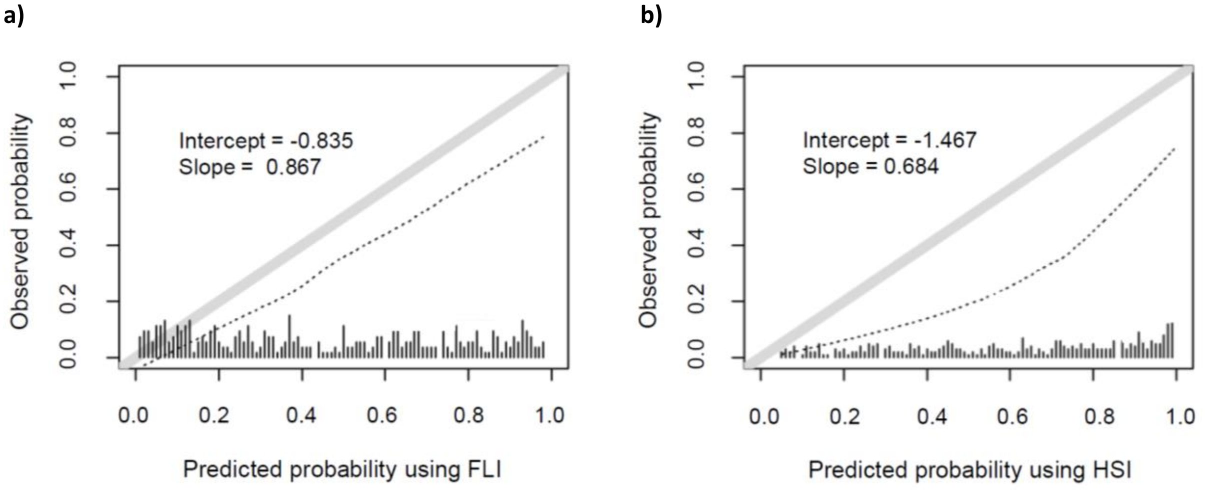

Supplement: ofae411_Supplementary_Data [file ofae411_supplementary_data.pdf]
